# Vegetation of *Ranunculus lateriflorus* (Ranunculaceae) in the Latorica (Latorytsia) River catchment (Slovakia and Ukraine)

**DOI:** 10.3897/BDJ.14.e189138

**Published:** 2026-05-11

**Authors:** Matej Dudáš, Lyubov Felbaba-Klushyna, Iryna Ivasyna, Ivana Slepáková, Richard Hrivnák

**Affiliations:** 1 P. J. Šafárik University, Košice, Slovakia P. J. Šafárik University Košice Slovakia; 2 Department of Botany, Uzhhorod National University, Uzhhorod, Ukraine Department of Botany, Uzhhorod National University Uzhhorod Ukraine; 3 Department of Plant Biology, Institute of Biology and Ecology, Pavol Jozef Šafárik University, Košice, Slovakia Department of Plant Biology, Institute of Biology and Ecology, Pavol Jozef Šafárik University Košice Slovakia; 4 Department of Microbiology, Institute of Biology and Ecology, Pavol Jozef Šafárik University, Košice, Slovakia Department of Microbiology, Institute of Biology and Ecology, Pavol Jozef Šafárik University Košice Slovakia; 5 Institute of Botany, Plant Science and Biodiversity Center, Slovak Academy of Sciences, Bratislava, Slovakia Institute of Botany, Plant Science and Biodiversity Center, Slovak Academy of Sciences Bratislava Slovakia

**Keywords:** field depression vegetation, *Isoëto-Nanojuncetea* Pannonian lowland, *Ranunculetum lateriflori*, vegetation affinity, wetland vegetation

## Abstract

Temporary pools and field depressions are unusual habitats that share features of both aquatic and terrestrial ecosystems. They often host rare vegetation determined by specific ecological conditions and harbour numerous threatened vascular plant species, such as *Ranunculus lateriflorus*, which is rare in the Pannonian lowland of Central Europe. We studied vegetation containing this species using a traditional phytosociological approach between 2023 and 2025 in the Latorica (Latorytsia) River catchment area in Slovakia and Ukraine (elevation range 95–105 m a.s.l.; mean annual temperature of 9.54–9.69°C; annual precipitation of 594–677 mm). In total, 91 vascular plant taxa were identified in 20 phytosociological relevés containing *R. lateriflorus*, which were classified into four clusters using TWINSPAN. We found that *R. lateriflorus* grows in stands of the *Eleocharito palustris–Sagittarion sagittifoliae* Passarge 1964 alliance (*Bidentetea* Tx. et al. ex. von Rochow 1951 class and mainly in the *Verbenion supinae* Slavnić 1951 alliance (class *Isoëto-Nanojuncetea* Br.-Bl. et Tx. in Br.-Bl. et al. 1952). In addition, the rare plant community *Ranunculetum lateriflori* association (*Verbenion supinae*) was recorded in both countries, for the first time in Ukraine.

## Introduction

**Figure 1.**
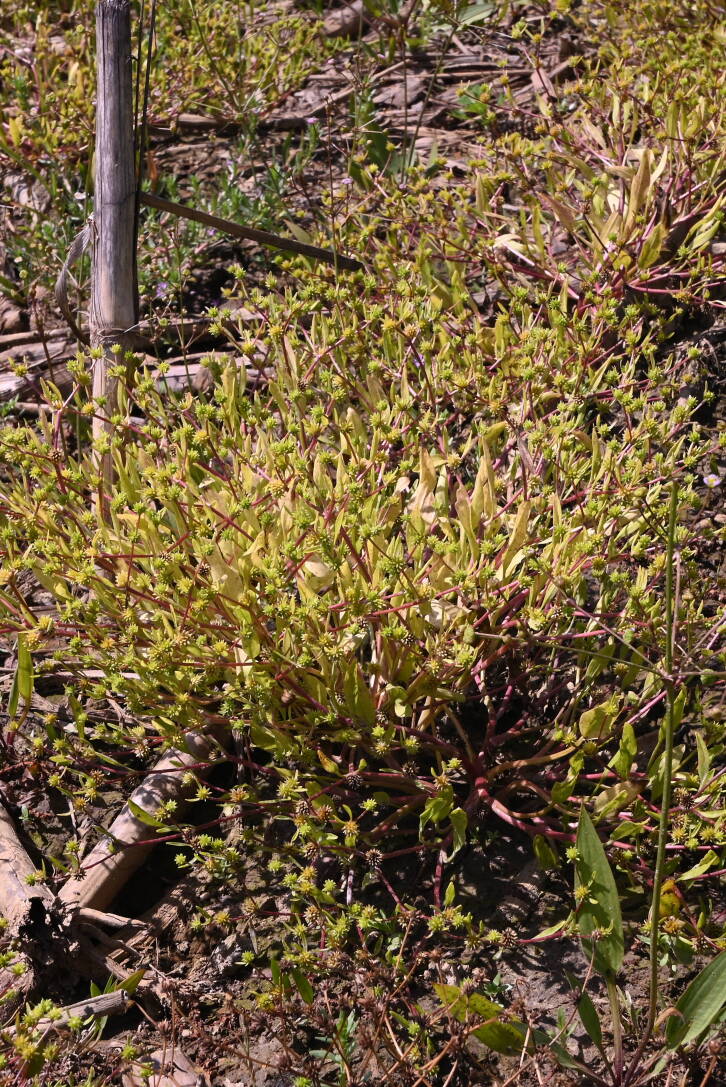
*Ranunculus lateriflorus*, Malý Horeš, R. Hrivnák.

Freshwater ecosystems are consistently at higher risk of degradation than their terrestrial or marine counterparts (Dudgeon et al. 2007). In Europe, freshwater habitats are represented by various vegetation types (Chytrý et al. 2020), several of which are considered threatened (Janssen et al. 2016). These habitats host numerous rare and endangered plant species that depend on specific ecological conditions and a limited level of anthropogenic disturbance for their survival. For example, pioneer ephemeral dwarf-cyperaceous vegetation, occurring in periodically flooded freshwater habitats with pronounced seasonal water-level fluctuations, ranks amongst the most endangered habitat types. Despite their vulnerability, these habitats often exhibit high species richness, with vascular plants listed in the IUCN and national Red Lists frequently serving as indicator and characteristic species of such vegetation types (Lukács et al. 2013, Janssen et al. 2016). In particular, field depressions experiencing spring flooding followed by a seasonal decline of the water table below the soil surface, combined with ongoing agricultural activities, represent highly specific habitats. These conditions are suitable for annual, competitively weak and rare plant species, such as *Ranunculus lateriflorus* DC. (Fig. 1). This species is distributed across Europe, except for its northern regions (Euro+Med PlantBase 2006). In Europe, the species is typical of pioneer ephemeral dwarf-cyperaceous vegetation in periodically freshwater-flooded habitats of Eurasia of the *Isoëto-Nanojuncetea* class (Chytrý et al. 2024), but it rarely occurs in saline vegetation (Borhidi et al. 2012). In Central Europe, the species is distributed along the Danube and Tisza Rivers in Hungary, Slovakia and Ukraine. The Tisza River region in Slovakia and Ukraine shows a strong concentration of records, including many from recent years, compared with other parts of the Central European range (GBIF 2026). In southern Slovakia, *R. lateriflorus* is rare and confined mainly to saline habitats and wetlands (Futák 1982, Kantor and Marhold 2024). In Ukraine, the species is distributed primarily in the eastern regions, while in the western part of the country, including the Transcarpathian Region, it is rare (GBIF 2026, Dudáš and Felbaba-Klushyna 2026).

The absence of recent vegetation data from eastern Slovakia (Valachovič et al. 2001), in contrast to recently recorded occurrences in Ukraine (Dudáš and Felbaba-Klushyna 2026), drew attention to vegetation with the presence of *R. lateriflorus*. Therefore, we aimed to study the habitats with the occurrence of *R. lateriflorus* in south-eastern Slovakia and south-western Ukraine within the Latorica (Latorytsia) River catchment, to characterise the vegetation affinity of the species in this region.

## Materials and Methods

**Figure 2.**
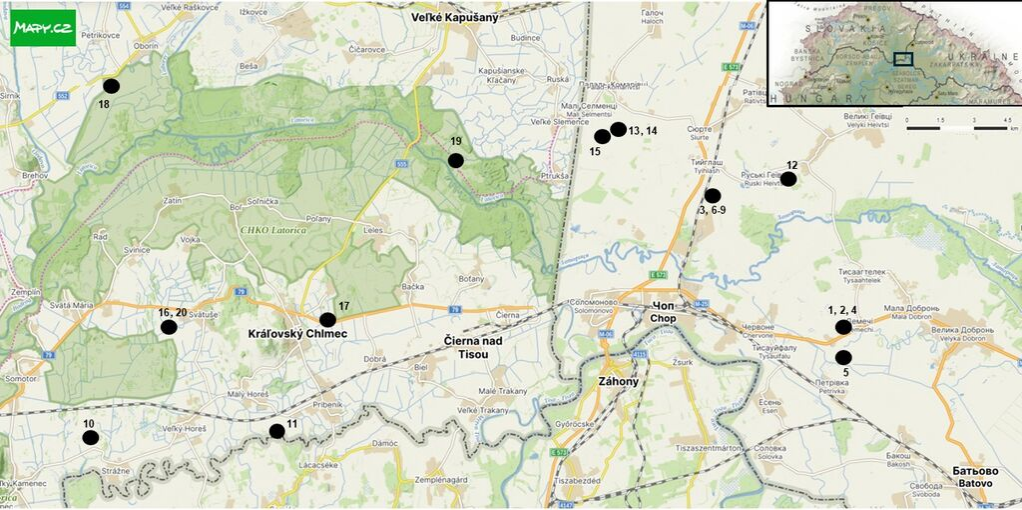
Study area with marked locations of phytosociological relevés; the black rectangle in the upper right corner of the map indicates the location of the study area along the Slovakia–Ukraine border within a broader geographical context. The numbering of the relevés corresponds to Table 1. Basemap: http://www.mapy.com.

The vegetation affinity of *Ranunculus lateriflorus* was analysed using phytosociological relevés recorded by the authors during the 2023–2025 vegetation seasons in the Latorica (Latorytsia) River catchment area in eastern Slovakia and western Ukraine (Fig. 2). All visually distinct stands containing *R. lateriflorus* were recorded within plots of 16 m². Phytosociological relevés were sampled following the traditional Zürich–Montpellier approach and the cover of plant species was visually estimated using the modified Braun–Blanquet scale (Dengler et al. 2008). In total, 20 relevés were obtained and stored in the Turboveg database (Hennekens and Schaminée 2001) and subsequently processed using the JUICE programme (Tichý 2002).

Species taxonomy was unified following the concept of broadly defined plant taxa, with a limited number of aggregate taxa, namely *Bolboschoenus maritimus* agg. (*B. laticarpus*, *B. planiculmis*, *Bolboschoenus* sp.), *Eleocharis palustris* agg. (*E. palustris*, *E. uniglumis*) and *Glyceria fluitans* agg. (*G. fluitans*, *G. notata*). Numerical classification was performed using the TWINSPAN algorithm (Hill 1979) to support the identification of variability in the species composition of the studied vegetation types. Two pseudospecies cut levels (0% and 50%) were applied to reflect the combined effects of species composition and dominance typical of many wetland vegetation types. Differential species for each cluster were identified, based on frequency and fidelity thresholds (Φ—phi coefficient; Chytrý et al. 2002), set at frequency ≥ 40%, phi coefficient ≥ 0.30 and a difference in frequency between clusters of ≥ 20%. Species that were constant (frequency ≥ 50%) in two or more clusters were not considered differential. Although the thresholds were set subjectively, such characteristics of diagnostic species are commonly used in vegetation studies, albeit with different values (e.g. Landucci et al. 2013, Douda et al. 2015). Fisher’s exact test (p < 0.05) was used to exclude species with non-significant occurrences in a given cluster (Tichý and Chytrý 2006).

Climatic data (mean annual temperature and annual precipitation) were obtained from the WorldClim database (Fick and Hijmans 2017).

The identification of the *Ranunculetum lateriflori* association was based on the formal definition presented by Šumberová and Hrivnák (2013), which follows the original description by Pop (1962). Hence, a relevé that contains at least two of the three diagnostic species (*Elatine alsinastrum*, *Ranunculus lateriflorus* and *R. polyphyllus*) of the "*Ranunculus lateriflorus*" sociological species group is considered to belong to the *Ranunculetum lateriflori* association (Šumberová and Hrivnák 2013).

The nomenclature of vascular plants and higher vegetation syntaxa follows the checklists of the online SlovPlantList database (Letz et al. 2024) and Mucina et al. (2016), respectively. Association names follow Šumberová and Hrivnák (2013). Rare and threatened species in Slovakia and Ukraine are listed according to Eliáš et al. (2015) and Didukh (2009), respectively.

## Results

All sampling plots were located in depressions within fields or along field roads, flooded by shallow water in spring, at elevations of 95–105 m a.s.l., with a mean annual temperature of 9.54–9.69°C (mean 9.63°C) and annual precipitation of 594–677 mm (mean 626 mm).

In total, 91 vascular plant taxa were identified in 20 phytosociological relevés containing *Ranunculus lateriflorus*. Apart from *R. lateriflorus*, the most frequent species in the dataset were *Alisma lanceolatum* and *R. sardous* (both with a frequency of 65%), followed by *Bidens frondosa*, *Lythrum hyssopifolia* and *Tripleurospermum inodorum* (all with a frequency of 60%) and *Ambrosia artemisiifolia* and *Echinochloa crus-galli* (both with a frequency of 55%). Nine species (*Elatine alsinastrum*, *Gratiola officinalis*, *Limosella aquatica*, *Lythrum hyssopifolia*, *Myosurus minimus*, *Ranunculus lateriflorus*, *R. peltatus*, *Rumex stenophyllus* and *Veronica scutellata*), recorded in the relevés from Slovakia, are included in the Slovak Red List, whereas no species from the Ukrainian Red Book were recorded in the relevés from Ukraine.

Four clusters were identified using TWINSPAN classification (Table 1 and Suppl. material 2).

**Figure 3.**
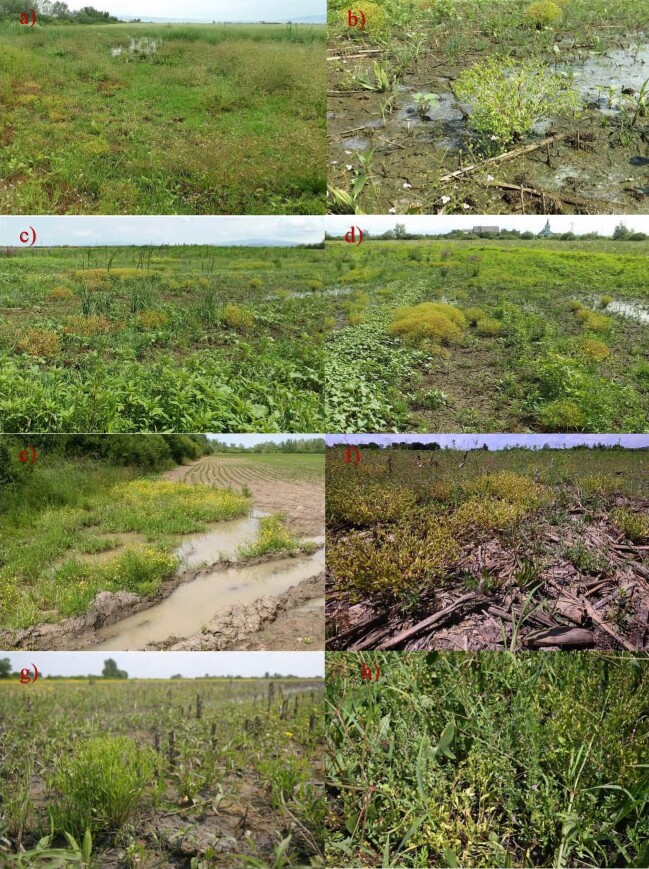
Plant communities with the *Ranunculus lateriflorus* in Slovakia and Ukraine: cluster 1, a) Tyihlash (Тийглаш) and b) Demechi (Демечі), author of both photographs M. Dudáš; cluster 2, c), d) Demechi (Демечі), M. Dudáš; cluster 3, e) Mali Selmentsi (Малі Селменці), M. Dudáš and f) Malý Horeš, R. Hrivnák; cluster 4, g) Svätuše, M. Dudáš and h) Kráľovský Chlmec, R. Hrivnák.

The first cluster comprises four relevés characterised by a high presence of species from the *Phragmito-Magnocaricetea* class (e.g. *Alisma lanceolatum*, *Bolboschoenus maritimus* agg., *Eleocharis palustris* agg. or *Oenanthe aquatica*) and, diagnostic species *Apera spica-venti*, *Butomus umbellatus*, *Lycopus europaeus* and a relatively low frequency of species from the *Isoëto-Nanojuncetea* class (Table 1, rels 1–4; Fig. 3a, b) with ~14 species per relevé. These relevés were classified within the *Eleocharito palustris>–Sagittarion sagittifoliae* alliance.

The second cluster, comprising five relevés, is differentiated by weeds as diagnostic species (e.g. *Echinochloa crus-galli* and *Xanthium italicum*), with co-dominance of *Bidens frondosa*. Species of the *Isoëto-Nanojuncetea* class are relatively well represented (Table 1, rels 5–9; Fig. 3c, d) with ~14 species per relevé. The relevés in this cluster were classified within the *Bidentetea* class, showing a transitional position towards the *Isoëto-Nanojuncetea* class.

The next two clusters contain a high proportion of *Isoëto-Nanojuncetea* species, but differ mainly in the presence of weed species. In addition to species typical of the exposed-bottom vegetation of *Isoëto-Nanojuncetea* class, the third cluster, comprising six relevés (Table 1, rels 10–15; Fig. 3e, f), includes several weeds, such as *Anagallis arvensis*, *Chenopodium polyspermum* and *Oxalis stricta*, as well as hygrophilous species, such as *Alisma lanceolatum*, *Persicaria hydropiper* and *Ranunculus flamulla*. This cluster was the species richest, with approximately 20 species per relevé. These relevés were classified as a ruderal variant of the *Verbenion supinae* alliance. The last (five relevés, ~16 per relevé; Table 1, rels 16–20; Fig. 3g, h) represent the typical variant of the *Verbenion supinae* alliance.

Relevés 15, 16, 19 and 20 (Table 1) meet the formal definition of the *Ranunculetum lateriflori* association due to the presence of *Elatine alsinastrum* and *Ranunculus lateriflorus* , as well as other diagnostic species of the *Isoëto-Nanojuncetea* class.

## Discussion

*Ranunculus lateriflorus* is generally considered a diagnostic species of the class *Isoëto-Nanojuncetea* and a diagnostic species of the EUNIS habitat R65 Continental subsaline alluvial pasture and meadow (Chytrý et al. 2024). However, its occurrence is much broader across wetland and saline vegetation types (Chytrý et al. 2020, Chytrý et al. 2024). A similar coenological pattern is typical of Central European landscapes, where the species is reported as characteristic and dominant in two plant communities, *Ranunculetum lateriflori* (Coldea 1997, Valachovič et al. 2001) and *Rorippo kerneri–Ranunculetum lateriflori* (Borhidi et al. 2012). According to the authors, the first association is characterised by diagnostic species of exposed-bottom vegetation of the *Isoëto-Nanojuncetea* class, whereas the second community comprises numerous halophytic species, lacks *Isoëto-Nanojuncetea* taxa and is thus assigned to the *Festuco-Puccinietalia* class. Our results show that the habitat range of the species in the studied area is broader, from vegetation of emergent helophytes on muddy soils of shallow streams and ponds with a fluctuating water table of temperate and boreal Eurasia (*Eleocharito palustris–Sagittarion sagittifoliae* alliance), across summer-annual pioneer vegetation of seasonally flooded nutrient-rich river alluvia, lacustrine banks and heavily nutrient-loaded anthropogenic habitats of boreo-temperate Europe and North Africa (*Bidentetea* class), to pioneer ephemeral dwarf-cyperaceous vegetation in periodically freshwater-flooded habitats of Eurasia (*Isoëto-Nanojuncetea* class). The habitat and climatic characteristics of localities with *R. lateriflorus* were similar due to the geographically small study area, limited to lowland field depressions in the Latorica (Latorytsia) River catchment. Despite the low variability of these factors and the small number of records, the species composition was relatively broad. This is likely due to variation in other, mainly local, ecological conditions, such as flood duration and water depth, the rate of water level decline, trophic status, substrate salinity and agricultural management, which are generally considered important for wetland vegetation (e.g. Deil 2005, Batzer and Sharitz 2014). For example, high water table and a prolonged period of the water level decline below the soil surface support the development of marsh vegetation of the *Phragmito-Magnocaricetea* class (Šumberová et al. 2011). A high nutrient content combined with relatively short flood duration and rapid water drawdown favours a high abundance of weeds and eutrophic marsh species typical of the *Bidentetea* class (e.g. Jarolímek et al. 1997). In contrast, long-term flooding with a shallow water-table, gradual water level decline, mesotrophic to slightly eutrophic soil conditions, absence of intensive agricultural management during the vegetation season and low fertilisation are suitable for the development of exposed-bottom vegetation of the *Isoëto-Nanojuncetea* class (Deil 2005). Therefore, based on the diverse environmental conditions in the studied area, we documented a relatively broad vegetation affinity of *Ranunculus lateriflorus*.

*Ranunculetum lateriflori* has been reported from Slovakia in previous surveys of exposed-bottom vegetation of the *Isoëto-Nanojuncetea* class (Valachovič et al. 2001, Šumberová and Hrivnák 2013). Only five and four relevés from the 1960s to the 1980s of the twentieth century were used in these syntaxonomical surveys, respectively. All relevés of the association presented here were collected in the south-eastern part of Slovakia, in the Východoslovenská nížina (Eastern Slovak) Lowland. The typical habitat comprises shallow field depressions that are flooded in spring and subsequently dry out at the beginning of summer (Husák and Oťaheľová 1985, Mochnacký 1988, Valachovič et al. 2001). Our data from recent years show that this community has persisted in this area over a long period. In contrast, the association has not been previously reported from Ukraine (Dubyna and Iemelianova 2019, Dubyna et al. 2021). Therefore, the relevé from Mali Selmentsi (Малі Селменці) Village represents the first documented phytosociological record of this association from Ukraine. The association *Ranunculetum lateriflori* is rare in Central Europe. Besides Slovakia, it has been reported only from Romania, specifically the Danube Valley and Transylvania (Coldea 1997), where it was originally described by Pop (1962). In Hungary, the association with a similar species composition, *Rorippo kerneri–Ranunculetum lateriflori* (Soó 1947) Borhidi 1996, has been reported, but it is characterised by a higher presence of halophyte species (Borhidi et al. 2012). This community has also been reported from the Tisza Region (Deák et al. 2009); however, we assume that *Ranunculetum lateriflori* may occur in Hungary, as well, due to the similar ecological conditions in the Latorica Region of Slovakia and Ukraine and in the adjacent Tisza River region.

*Ranunculus lateriflorus* is considered a rare and endangered species in Slovakia (Eliáš et al. 2015), classified as Vulnerable. In the Transcarpathian region of Ukraine, it was long considered extinct (Hamor et al. 2009), but was recently re-discovered (Dudáš and Felbaba-Klushyna 2026). Based on recent findings in western Ukraine, the species has also been classified as Endangered (EN; Dudáš and Felbaba-Klushyna 2026).

## Conclusion

Based on 20 phytosociological relevés, we documented the vegetation affinity of *Ranunculus lateriflorus* in the Latorica (Latorytsia) River catchment. All stands were found in fields flooded by shallow water in spring, which is a key prerequisite for the occurrence of the species in the studied area. We showed that the species occurs in plant communities belonging to the *Phragmito-Magnocaricetea*, *Bidentetea* and *Isoëto- Nanojuncetea* classes, with increasing species richness of the stands in this order. In addition, the rare plant community *Ranunculetum lateriflori* was confirmed in both countries and for the first time in Ukraine.

## Supplementary Material

7937C69A-CE8F-5AEF-B97B-5C50F0A417B6 10.3897/BDJ.14.e189138.suppl1 Supplementary material 1 Species with one occurrence Data type doc Brief description Species with occurrence only in one relevé and localities of relevés presented in Table 1. File: oo_1541928.docx https://binary.pensoft.net/file/1541928 Matej Dudáš & Richard Hrivnák

137A517C-2118-555B-9C5E-91CF96EA17D4 10.3897/BDJ.14.e189138.suppl2 Supplementary material 2 Classification tree Data type doc Brief description Classification tree produced by the TWINSPAN algorithm (clusters correspond to Table 1 in the main text). File: oo_1562963.docx https://binary.pensoft.net/file/1562963 Richard Hrivnák

## Figures and Tables

**Table 1.** Phytosociological relevés with *Ranunculus lateriflorus* (BI – *Bidentetea*, IN – *Isoëto-Nanojuncetea*, PM – *Phragmito-Magnocaricetea*; cluster 1: relevés 1–4, 2: 5–9, 3: 10–15, 4: 16–20). Species with occurrence only in one relevé and localities of relevés in Table 1 (a = 2a, b = 2b) are presented in Suppl. material 1.

**Class / Taxon / Relevé number**		**1**	**2**	**3**	**4**	**5**	**6**	**7**	**8**	**9**	**10**	**11**	**12**	**13**	**14**	**15**	**16**	**17**	**18**	**19**	**20**
	**Diagnostic species of the first cluster**																				
PM	*Lycopus europaeus*	+	**r**	.	+	.	.	.	.	.	+	.	.	.	.	.	.	.	.	.	.
	*Apera spica-venti*	**r**	+	.	.	.	.	.	.	.	.	.	.	.	.	.	.	.	.	.	.
PM	*Butomus umbellatus*	**r**	.	.	**r**	.	.	.	.	.	.	.	.	.	.	.	.	.	.	.	.
	**Diagnostic species of the second cluster**																				
BI	*Xanthium italicum*	a	b	.	+	**a**	**a**	**a**	**a**	**1**	.	.	.	+	.	.	.	.	.	.	.
	*Echinochloa crus-galli*	+	+	1	.	**1**	**b**	**b**	**3**	**a**	.	.	.	a	4	b	.	.	.	.	.
	**Diagnostic species of the third cluster**																				
IN	*Peplis portula*	.	r	.	.	.	.	.	.	.	.	**1**	**a**	**1**	+	**r**	.	.	.	.	.
	*Anagallis arvensis*	.	.	.	.	.	.	.	.	.	.	.	.	**r**	+	+	.	.	.	.	.
	*Ranunculus flammula*	.	.	.	.	.	.	.	.	.	+	**r**	+	**r**	.	.	.	.	.	.	.
IN	*Juncus bufonius*	.	.	r	.	.	.	.	.	.	.	+	**1**	**1**	**1**	**1**	+	1	.	+	.
BI	*Chenopodium polyspermum*	.	.	.	.	.	.	.	r	.	.	.	.	**r**	+	**r**	.	.	.	.	.
	**Diagnostic species of the fourth cluster**																				
IN	*Myosurus minimus*	.	.	.	.	.	.	.	.	.	.	.	.	.	.	r	+	.	+	.	**a**
	*Elatine alsinastrum*	.	.	.	.	.	.	.	.	.	.	.	.	.	.	r	+	.	.	**r**	**a**
PM	*Alisma plantago-aquatica*	.	.	+	1	.	.	.	.	.	.	.	.	.	.	+	.	+	+	**a**	**a**
	**Common taxa for the first two clusters**																				
	*Veronica scutellata*	**b**	**r**	**r**	**1**	.	**r**	+	+	**a**	+	.	.	.	.	.	.	.	.	.	.
BI	*Bidens frondosa*	**a**	**1**	+	+	**3**	**a**	**1**	**a**	**3**	+	r	.	r	.	.	.	.	.	.	.
PM	*Eleocharis palustris* agg.	+	**3**	.	.	**a**	.	.	.	+	.	.	.	.	.	.	.	+	.	.	.
	**Common species for the second two clusters**																				
	*Polygonum aviculare*	.	r	.	.	.	.	.	.	.	+	.	.	**r**	+	+	**r**	+	+	.	.
IN	*Limosella aquatica*	.	.	.	.	.	.	.	.	.	.	+	.	+	.	+	+	+	.	.	**r**
IN	*Ranunculus sardous*	.	.	.	.	.	r	+	.	r	**a**	.	**r**	**r**	**r**	**b**	**b**	+	+	+	+
	***Isoëto-Nanojuncetea***																				
	*Ranunculus lateriflorus*	1	+	+	+	+	b	b	a	a	+	3	+	3	+	1	+	a	r	a	+
	*Lythrum hyssopifolia*	.	.	r	.	+	+	r	r	.	.	+	1	1	b	+	3	a	.	.	.
	*Gnaphalium uliginosum*	.	+	.	r	.	r	r	+	.	.	+	.	+	r	.	+	.	1	.	.
	*Plantago uliginosa*	.	.	.	.	.	+	r	r	.	.	r	.	.	+	r	.	.	.	.	.
	***Phragmito-Magnocaricetea***																				
	*Alisma lanceolatum*	1	+	4	+	a	1	a	1	.	+	1	.	+	.	+	.	a	.	.	.
	*Oenanthe aquatica*	.	.	r	r	.	.	.	.	1	.	.	.	.	.	.	.	.	.	.	.
	*Glyceria fluitans* agg.	.	+	.	.	.	.	.	r	.	.	.	.	.	.	.	.	.	.	+	.
	*Bolboschoenus maritimus* agg.	+	.	.	a	.	.	.	.	.	.	.	.	.	.	.	.	.	+	.	.
	***Bidentetea tripartitae***																				
	*Persicaria hydropiper*	.	.	.	.	.	.	.	.	.	+	.	.	r	1	.	.	.	.	.	.
	*Alopecurus aequalis*	.	.	.	.	.	.	.	.	.	.	.	.	.	.	.	a	+	.	.	.
	**Other species with occurrence in at least two relevés**																				
	*Tripleurospermum inodorum*	.	.	.	.	.	r	.	+	+	+	+	.	+	r	r	+	+	+	r	.
	*Ambrosia artemisiifolia*	.	+	.	.	.	r	.	+	1	.	+	a	.	r	+	+	a	.	1	.
	*Ranunculus sceleratus*	.	.	1	.	.	+	.	r	.	+	+	r	.	.	r	.	.	r	a	r
	*Alopecurus geniculatus*	.	r	.	.	r	.	.	.	.	a	.	.	+	.	r	.	.	+	+	r
	*Rorippa sylvestris*	.	.	.	.	r	.	r	r	.	.	.	.	.	.	.	a	+	.	.	.
	*Persicaria lapathifolia*	r	r	.	.	.	.	.	.	.	.	.	.	.	r	+	.	+	.	.	.
	*Lythrum salicaria*	.	r	.	.	.	.	.	.	.	.	.	r	.	.	.	.	+	r	.	.
	*Setaria pumila*	.	.	.	.	.	.	.	.	b	.	1	.	.	.	.	a	+	.	.	.
	*Callitriche* sp.	.	.	.	.	.	.	.	.	.	.	r	+	.	.	.	.	.	.	+	r
	*Ranunculus repens*	r	r	.	.	.	.	.	.	.	.	.	r	+	.	.	.	.	.	.	.
	*Juncus effusus*	.	.	.	.	+	.	.	.	.	.	r	.	.	.	.	.	.	.	r	.
	*Ranunculus peltatus*	.	.	.	.	.	.	.	.	.	.	.	.	.	.	.	.	.	r	r	.
	*Oxalis stricta*	.	.	.	.	.	.	.	.	.	.	.	.	r	+	.	.	.	.	.	.
	*Trifolium* sp.	.	.	.	.	.	.	.	.	.	.	.	.	.	r	.	+	.	.	.	.
	*Lolium perenne*	.	.	.	.	.	.	.	.	.	+	r	.	.	.	r	.	.	.	.	.
	*Gratiola officinalis*	.	.	.	.	.	.	.	.	.	.	+	r	.	.	.	.	.	.	.	.
	*Poa trivialis*	.	.	.	.	.	.	.	.	.	+	.	.	.	.	.	.	.	.	+	.
	*Plantago major*	.	.	.	.	.	.	.	.	.	.	.	.	.	.	.	.	+	+	.	.
	*Capsella bursa-pastoris*	.	.	.	.	.	.	.	.	.	.	.	.	.	.	.	.	r	.	r	.
	*Galium palustre*	.	.	.	.	r	.	.	.	+	.	.	.	.	.	.	.	.	.	.	.
	*Spergula arvensis*	.	.	.	.	.	.	.	.	.	.	.	.	r	r	.	.	.	.	.	.
	*Juncus articulatus*	.	.	.	.	r	.	.	.	.	.	r	.	.	.	.	.	.	.	.	.
	*Rumex stenophyllus*	.	.	.	.	.	.	.	.	1	.	.	.	.	.	.	.	+	.	.	.
	*Lactuca serriola*	.	.	.	.	.	.	.	.	r	.	r	.	.	.	.	.	.	.	.	.
	*Lythrum virgatum*	r	.	.	.	.	.	.	r	.	.	.	.	.	.	.	.	.	.	.	.
	*Stachys palustris*	.	.	.	.	+	.	.	.	.	.	r	.	.	.	.	.	.	.	.	.
	*Persicaria amphibia*	.	.	.	.	.	.	.	.	.	.	.	.	.	.	.	.	.	+	.	+
	*Rorippa palustris*	.	r	.	.	.	.	.	.	.	.	.	.	.	.	.	.	.	.	r	.
