# Supplementary material for: Vegetation of Ranunculus lateriflorus (Ranunculaceae) in the Latorica (Latorytsia) River catchment (Slovakia and Ukraine)
Source: Biodivers Data J. 2026 May 11;14:e189138. doi: 10.3897/BDJ.14.e189138 (PMC13184627; doi:10.3897/BDJ.14.e189138)
Supplement: Supplementary material 1 — Species with one occurrence [file bdj-14-e189138-s001.docx]

**Suppl. material 1: Species with occurrence only in one relevé and localities of relevés presented in Table 1.**

Species with occurrence only in one relevé

*Abutilon theophrasti* relevé 18: cover r; *Agrostis stolonifera* 15: +; *Alopecurus pratensis* 10: +; *Bidens tripartitus* 10: +; *Brassica napus* 9: r; *Cardamine pratensis* agg. 10: +; *Chara*  sp. 17: +; *Cirsium arvense* 15: r; *Conyza canadensis* 11: r; *Erigeron annuus* 8: r; *Fallopia convolvulus* 5: r; *Galium palustre* 10: +; *Iva xanthiifolia* 12: a; *Juncus* cf. *gerardii* 18: r; *Lactuca saligna* 18: r; *Lemna minor* 3: 1; *Lycopus exaltatus* 4: +; *Matricaria chamomilla* 19: +; *Mentha aquatica* 2: r; *M. pulegium* 10: +; *Poa annua* 10: +; *P. palustris* 10: +; *Populus alba* 14: r; *Potentilla reptans* 2: r; *Rumex* crispus 10: +; *R. palustris* 18:+; *Sonchus asper* 9: r; *Trifolium repens* 10: +; *Typha latifolia* 5: r; *Veronica anagallis-aquatica* 20: r; *V. serpylifolia* 10: +; *Viola arvensis* 17: r.

Localities of relevés

1. Western Ukraine (WU), Demechi (Демечі), wet field depression on the W edge of the village; Latitude: 48°25'26.584"N; Longitude: 22°19'13.380"E; altitude: 100 m; area 16 m^2^, cover E1: 85 %; Date: 9. 6. 2024; Authors: M. Dudáš & L. Felbaba-Klushyna.

2. WU, Demechi (Демечі), wet field depression on the W edge of the village; 48°25'25.430"N; 22°19'11.912"E; 100; 16; 75; 9. 6. 2024; M. Dudáš & L. Felbaba-Klushyna.

3. WU, Tyihlash (Тийглаш), field depression on SE edge of village; 48°28'35.147"N; 22°13'55.206"E; 100; 16; 60; 9. 6. 2024; M. Dudáš & L. Felbaba-Klushyna.

4. WU, Demechi (Демечі), wet field depression on the W edge of the village; 48°25'25.174"N; 22°19'12.299"E; 100; 16; 80; 9. 6. 2024; M. Dudáš & L. Felbaba-Klushyna.

5. WU, Zakarpattia Oblast, Prytysyanska lowland, Demechi (Демечі), 1.5 km S from the village, wet field depression with high coverage of organic materiál (90 %); 103; 48°24'53.132"N; 22°19'33.039"E; 100; 16; 9. 6. 2025; M. Dudáš, L. Felbaba-Klushyna & I. Ivasyna.

6. WU, Tyihlash (Тийглаш), field depression on SE edge of village; 48°28'36.310"N; 22°13'53.575"E; 100; 16; 65; 9. 6. 2024; M. Dudáš & L. Felbaba-Klushyna.

7. WU, Tyihlash(Тийглаш), field depression on SE edge of village, E from railway embankment; 48°28'35.188"N; 22°13'53.807"E; 101; 16; 70; 2. 6. 2024; M. Dudáš.

8. WU, Tyihlash (Тийглаш), field depression on SE edge of village; 48°28'34.698"N; 22°13'54.804"E; 100; 16; 90; 9. 6. 2024; M. Dudáš & L. Felbaba-Klushyna.

9. WU, Zakarpattia Oblast, Prytysyanska lowland, Tyihlash (Тийглаш), field depression on SE edge of village, E from railway embankment; 48°28'35.147"N; 22°13'55.206"E; 101; 16; 100; 9. 6. 2025; M. Dudáš, L. Felbaba-Klushyna & I. Ivasyna.

10. Eastern Slovakia (ES), Východoslovenská nížina Lowland, Strážné, Opátske piesky, field depression N from the village; 48°22'55.661"N; 21°51'26.885"E; 95; 16; 65; 6. 6. 2024; R. Hrivnák & M. Dudáš.

11. ES, Východoslovenská nížina Lowland, Malý Horeš, 0.9 km SE from collective farm on the SE edge of the village, field depression with temporary water pool on corn field; 48°23'16.50"N; 21°58'14.65"E; 97; 16; 70; 4. 6. 2025; M. Dudáš & I. Ivasyna.

12. WU, Zakarpattia Oblast, Prytysyanska lowland, Mali Heivtsi (Малі Геївці), field depression 0,5 km SW from the village with standing water after storm; 48°29'29.699"N; 22°17'21.397"E; 100; 16; 85; 23. 5. 2023; M. Dudáš.

13. WU, Zakarpattia Oblast, Prytysyanska lowland, Mali Selmentsi (Малі Селменці), 2.8 km SE from the village, wet edge of the field with soybean; 48°29'34.79"N; 22°10'38.23"E; 105; 16; 70; 9. 6. 2025; M. Dudáš, L. Felbaba-Klushyna & I. Ivasyna.

14. WU, Mali Selmentsi, 2,8 km SSE from the village, wet edge of the field with soybean; 48°29'34.232"N; 22°10'37.230"E; 105; 16; 70; 9. 6. 2025; M. Dudáš, L. Felbaba-Klushyna & I. Ivasyna.

15. WU, Mali Selmentsi, 2.85 km SSE from the village, wet edge of the field; 48°29'30.463"N; 22°10'32.183"E; 105; 16; 50; 9. 6. 2025; M. Dudáš, L. Felbaba-Klushyna & I. Ivasyna.

16. ES, Východoslovenská nížina Lowland, Svätuše, wet edge of the grain field ca 1 km W from village; 48°25'50.25"N; 21°54'5.94"E; 100; 16; 85; 4. 6. 2025; M. Dudáš & I. Ivasyna.

17. ES, Východoslovenská nížina Lowland, Kráľovský Chlmec, 1.3 km S from settlement Fejséš, field depression with temporary water pool under road no. 79; 48°25'38.46"N; 21°59'39.67"E; 104; 16; 80; 4. 6. 2025; M. Dudáš & I. Ivasyna.

18. ES, Východoslovenská nížina Lowland, Kucany, field depression with temporary water pool, ca 1.3 km W from the village; 48°31'41.297"N; 21°51'24.199"E; 100; 16; 40; 23. 6. 2023, M. Dudáš.

19. ES, Východoslovenská nížina Lowland, Leles, muddy field road under southern river dam of Latorica River, 5 km N from the village; 48°30'29.797"N; 22°1'47.298"E; 100; 16; 95; 23. 5. 2023; M. Dudáš.

20. ES, Východoslovenská nížina Lowland, Svätuše, field depression with temporary water pool on corn field after harvest, ca 2.2 km W from the village; 48°25'50.19"N; 21°53'50.95"E; 98; 16; 50; 28. 5. 2023; M. Dudáš.
