# Supplementary material for: Vegetation of Ranunculus lateriflorus (Ranunculaceae) in the Latorica (Latorytsia) River catchment (Slovakia and Ukraine)
Source: Biodivers Data J. 2026 May 11;14:e189138. doi: 10.3897/BDJ.14.e189138 (PMC13184627; doi:10.3897/BDJ.14.e189138)

**Suppl. Material 2: Classification tree produced by the TWINSPAN algorithm (clusters correspond to Table 1 in the main text).**


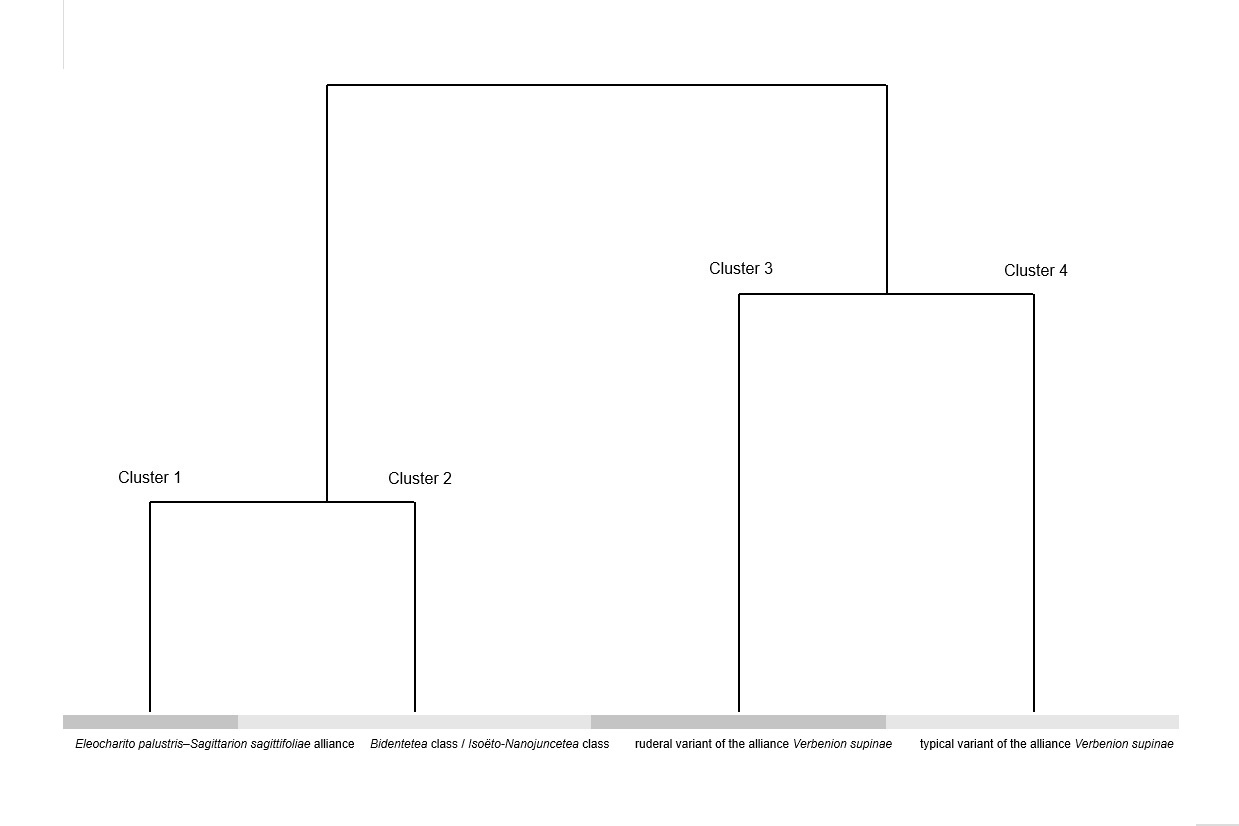

Supplement: Supplementary material 2 — Classification tree [file bdj-14-e189138-s002.docx]
